# Measurement of Recent Exposure to *Phlebotomus argentipes*, the Vector of Indian Visceral Leishmaniasis, by Using Human Antibody Responses to Sand Fly Saliva

**DOI:** 10.4269/ajtmh.2010.09-0336

**Published:** 2010-05

**Authors:** Meredith F. Clements, Kamlesh Gidwani, Rajiv Kumar, Jitka Hostomska, Diwakar S. Dinesh, Vijay Kumar, Pradeep Das, Ingrid Müller, Gordon Hamilton, Vera Volfova, Marleen Boelaert, Murari Das, Suman Rijal, Albert Picado, Petr Volf, Shyam Sundar, Clive R. Davies, Matthew E. Rogers

**Affiliations:** London School of Hygiene and Tropical Medicine, London, United Kingdom; Banaras Hindu University, Varanasi, India; Charles University, Prague, Czech Republic; Rajendra Memorial Research Institute, Patna, India; Imperial College of Science, Technology and Medicine, London, United Kingdom; Keele University, Keele, United Kingdom; Institute of Tropical Medicine, Antwerp, Belgium; BP Koirala Institute of Health Sciences, Dharan, Nepal

## Abstract

Antibody (IgG) responses to the saliva of *Phlebotomus argentipes* were investigated using serum samples from regions of India endemic and non-endemic for visceral leishmaniasis (VL). By pre-adsorbing the sera against the saliva of the competing human-biting but non-VL vector *P. papatasi*, we significantly improved the specificity of a *P. argentipes* saliva enzyme-linked immunosorbent assay. Using this method, we observed a statistically significant correlation between antibodies to *P. argenitpes* saliva and the average indoor density of female sand flies. Additionally, the method was able to detect recent changes in vector exposure when sera from VL patients were assayed before, during, and after hospitalization and protected from sand fly bites under untreated bed nets. Collectively, these results highlight the utility of antibodies to *P. argentipes* saliva as an important tool to evaluate VL vector control programs.

## Introduction

Visceral leishmaniasis (VL) in the Indian subcontinent, also known as kala-azar, is caused by *Leishmania donovani*. The disease is fatal if not treated, and VL is responsible for an estimated 60,000 deaths per year worldwide.^1^ The annual incidence of kala-azar cases is estimated to be 0.5 million and the prevalence to be 2.5 million.^1^ More than 90% of the world's reported VL cases are in India, Nepal, Bangladesh, Sudan, and Brazil. In India the main endemic focus is in Bihar State where resistance to the first-line treatment, pentavalent antimonials, is rife; there is no vaccine.^2^ In India, Nepal, and Bangladesh, *L. donovani* is transmitted exclusively by *Phlebotomus argentipes*, a sand fly that is considered anthroponotic.^3^,^4^ Because VL in the Indian subcontinent cycles predominantly between humans, with no established zoonotic involvement, control strategies have focused on prevention of infection through the promotion of early diagnosis and vector control.^5^,^6^

Currently, one of the biggest limitations to the evaluation of vector control programs is the accurate estimation of exposure to vector bites and transmission. Traditional methods rely heavily upon efficient trapping of vectors, and techniques such as human landing studies are now considered unethical for measuring protection in regions of endemicity.^7^,^8^ Field studies using enzyme-linked immunosorbent assay (ELISA) techniques have demonstrated the presence of antibodies to sand fly saliva in humans,^9^–^12^ foxes, and dogs^12^ naturally exposed to sand fly bites. Accordingly, these findings have stimulated interest in the use of antibodies against sand fly saliva as a diagnostic tool for evaluating exposure and/or risk of infection among humans living in areas endemic for leishmaniasis. However, the utility of a sand fly saliva ELISA has not been formally demonstrated in human populations in VL foci. The vector specificity of the technique and its sensitivity to reflect levels of exposure in humans has not been addressed. Furthermore, basic information on the persistence of human antibodies against sand fly saliva in the field is unknown. This is particularly important if the technique is to be used to estimate recent histories of sand fly bite.

The aim of this study was to investigate the potential utility of a novel *P*. *argentipes* saliva ELISA to estimate recent exposure of human populations to sand fly bite, and to obtain much needed information on the kinetics of human antibody responses to this important VL vector.

## Materials and Methods

### Study population.

We tested 240 serum samples that were divided into five main groups: 1) 62 samples from healthy persons living in a rural VL-endemic foci of Bihar state, India; 2) 27 healthy Indian controls from urban, non-VL areas of Western Uttar Pradesh (Indian non-endemic controls [NECs]); 3) 12 samples from residents in the United Kingdom with no history of travel to India, Nepal, or Bangladesh (UK NECs); 4) 52 VL patients with active kala-azar from Bihar (*L. donovani*-positive by spleen biopsy) re-sampled after their 30-day treatment in a hospital and 16 of these re-sampled patients six months after discharge from a hospital; and 5) 87 Indian and Nepalese samples from control households of the KALANET Long Lasting Insecticide Bed Net study (ClinicalTrials.gov CT-2005-015374) in which untreated bed nets were used.

Serum samples used in the study were obtained from patients ≥ 15 years of age (India and Nepal: age range = 15–62 years, median = 30 years; UK: age range = 21–35 years, median = 24 years). Patients with VL were treated with either amphotericin B or paromomycin and confined to the Kala-Azar Medical Research Center in Muzzafarpur, Bihar State. During this time, they were protected from additional sand fly bites by the personal use of untreated bed nets in a hospital that routinely sprays their grounds with insecticides. Ethical clearance for the study was obtained from the institutional review boards of B. P. Koirala Institute of Health Sciences, Dharan, Nepal, and the Institute of Medical Sciences, Banaras Hindu University, India. Written informed consent was obtained from all study participants and their households.

### Sand fly survey.

The abundance of sand flies was estimated for 15 and 16 months in households in Nepal and India, respectively. Twenty-five houses were randomly selected from each of five control clusters (three in India and two in Nepal) of the KALANET project in which there was no prior history or current use of bed nets. Each house was sampled for one night in September 2006 for *P*. *argentipes* by aspiration, and 10 households from each cluster with the highest sand fly density were selected for a complete entomologic survey. For each survey, a CDC light trap (Miniature Incandescent Light Trap Model 1012; J.W. Hock Company, Gainesville, FL) was used. Collection was performed during one night per month (6:00 pm to 6:00 am) from September 2006 to November and December 2007 in Nepal and India, respectively. One CDC light trap per house was installed typically near the corner of a bedroom situated 15 cm above the ground and 3 cm away from the wall. Collected sand flies were sexed and speciated under a binocular microscope. Geometric means of female *P*. *argentipes* sand flies were calculated per household (aggregate = 15 months) to evaluate the in-house density (as a proxy for exposure to sand flies). Sera from permanent adult occupants were collected from each household during November–December 2007 at the end of entomologic monitoring.

### Sand fly saliva preparation.

Saliva from colonized *P*. *argentipes* (Rajendra Memorial Research Institute, Patna, India, and Keele University, Keele, United Kingdom) or *P*. *papatasi* (Charles University, Prague, Czech Republic) was collected from female flies five days old post-emergence and maintained on 70% sucrose solution given *ad libitum*. Pools of 10 sand fly salivary gland pairs were collected in 50 μL of phosphate-buffered saline (PBS) on ice and individually pierced to release their saliva. After centrifugation (1,800 × *g* for 5 minutes), the saliva was collected from the supernatant fraction, leaving behind the salivary gland epithelia and other sand fly debris. Aliquots of saliva from 50 flies were collected, pooled, and frozen at −70°C until used. For transportation to the field, saliva was lyophilized and reconstituted in its original volume of distilled water for one hour at room temperature before use.

### Serologic analysis of antibodies against saliva.

Total specific IgG against saliva was measured by ELISA. All chemicals and reagents were obtained from Sigma (Irving, United Kingdom) unless otherwise stated. Microtiter plates (Maxisorb; Nunc, Roskilde, Denmark) were coated with 50 μL of 50 ng of *P*. *papatasi* saliva in 0.01 M carbonate-bicarbonate buffer, pH 9.6, overnight at 4°C. The wells were washed four times with 200 μL of PBS buffer containing 0.05% Tween 20 (PBS-Tween) and blocked with 200 μL of 5% bovine serum albumin (BSA) in PBS-Tween for 2 hours at 37°C. After washing, 100 μL of human sera were diluted 1:50 in PBS-Tween and incubated in duplicate overnight at 4°C (pre-adsorption step). In parallel, new plates were coated with 50 μL of 50 ng of *P*. *argentipes* saliva as described above and incubated overnight at 4°C without shaking. After washing and blocking of the *P. argentipes*-coated plates, sera were transferred from the *P*. *papatasi* plates to the *P*. *argentipes* plates and incubated for 2 hours at 37°C. The *P*. *papatasi* plates were washed, filled with 200 μL of wash buffer, sealed with parafilm, and kept at 4°C for 2 hours, thus enabling sufficient time for the *P*. *argentipes* and *P*. *papatasi* plates to reach the same point in the protocol and be processed together for the rest of the assay. One hundred microliters of biotinylated anti-human IgG was added at a dilution of 1:2,000 in PBS-Tween to all plates and incubated for 1 hour at 25°C. The plates were then washed and incubated with 100 μL of streptavidin-conjugated alkaline phosphatase at a dilution of 1:1,000. One hundred microliters of paranitrophenylphosphate (1 mg/mL) diluted in substrate buffer (carbonate-bicarbonate buffer with 10% diethanolamine and 3 mM MgCl_2_, pH 9.6) was added as a substrate and the absorbance of the yellow product was measured at 405 nm using a Spectramax 190 ELISA plate reader (Molecular Devices, Sunnyvale, CA) after 20 minutes of color development at 25°C in the dark. The cut-off value was set at two standard deviations from the mean of the Indian non-endemic control group.

### Antibody half-life.

The half-life of antibodies against salivary in patients was determined from VL hospitalization study data by using the equation half life (in days) = elapsed time × log^2^/log (average day 0 titer/average day 30 titer).

### Statistical analysis.

The ELISA data were not normally distributed. Therefore, the Mann-Whitney test was used to compare means using the Prism version 5 software (GraphPad Software, San Diego, CA). Correlations were tested by using the Spearman rank correlation coefficient. For those paired data in which patients were assayed over time, a Wilcoxon matched pairs test was used. A *P* value < 0.05 was considered significant.

## Results

**Figure 1.**
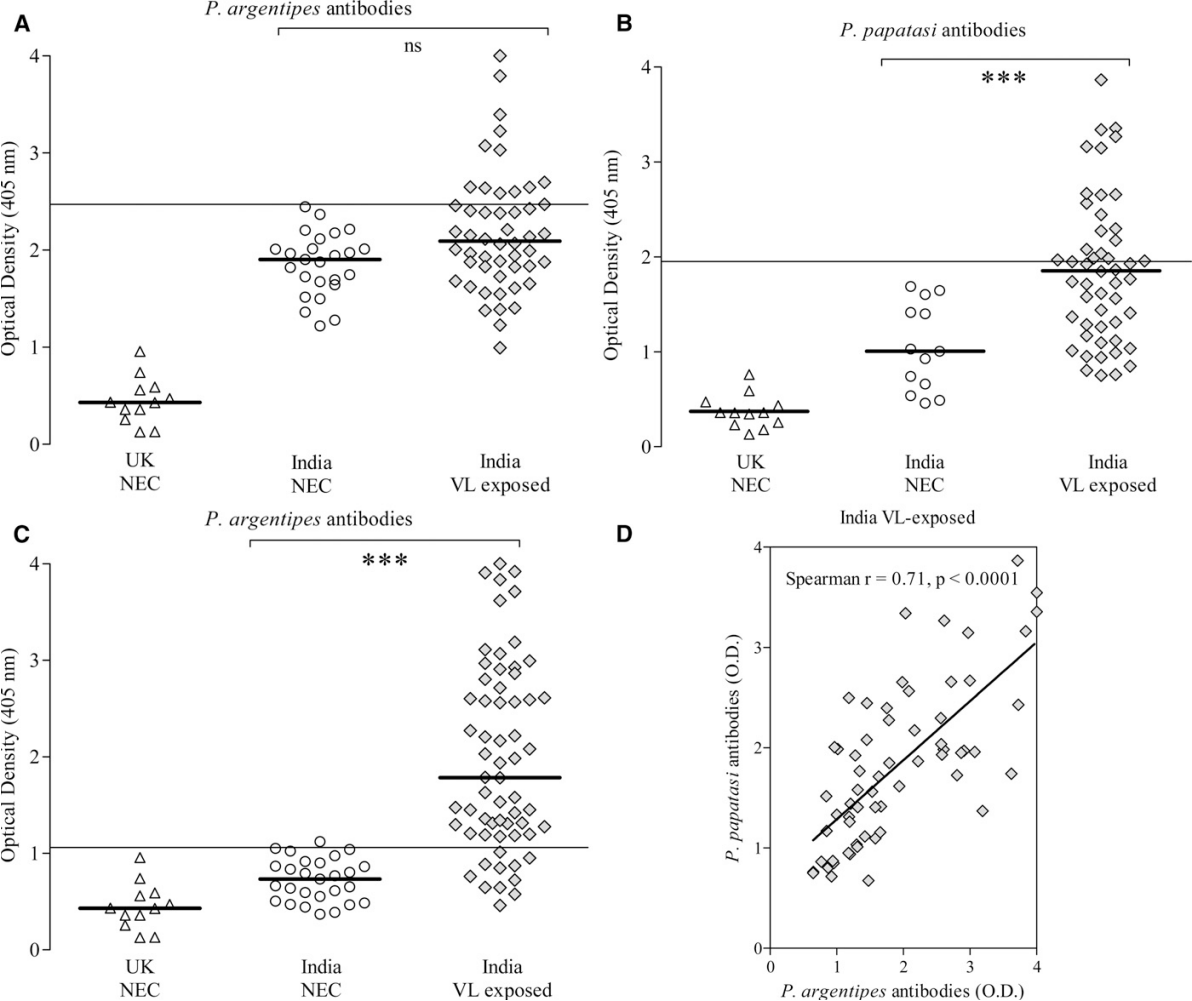
Human IgG response to *Phlebotomus argentipes* and *P. papatasi* saliva. Comparison of United Kingdom (open triangles, n = 12) and Indian non-endemic control (NEC) (open circles, unadsorbed, n = 25 and pre-adsorbed, n = 27) sera against sera from an area of India endemic for visceral leishmaniasis (VL) (filled diamonds, unadsorbed, n = 52 and pre-adsorbed, n = 62). Sera were processed for antibodies to *P. argentipes* (**A**) and *P. papatasi* (**B**) saliva by using a standard indirect enzyme-linked immunosorbent assay (ELISA) format. (**C**) Levels of antibodies against *P. argentipes* saliva by modified ELISA with a *P. papatasi* saliva pre-adsorption step. (**D**) Correlation between *P. argentipes* and *P. papatasi* saliva antibody responses from the Indian VL-exposed cohort. Bars in dot plots represent the median value of the groups. Lines represent cut-off values two standard deviations from the mean of the NEC group. Asterisks indicate statistical significance between the groups indicated (ns = not significant; ***P < 0.0005).

To investigate whether a human population from an leishmaniasis-endemic region had IgG against salivary gland antigens of the vector sand fly *P*. *argentipes*, we measured by ELISA the antibody level in sera of 89 adults from India and 12 adults from the United Kingdom. Analysis showed a high level of antibodies against *P*. *argentipes* saliva in persons from India compared with persons from the United Kingdom (*P* < 0.0001) (Figure 1A). The indirect ELISA used in all similar sand fly saliva studies to date did not detect any differences in antibody levels between non-endemic and leishmaniasis-endemic regions of India (*P* = 0.756) (Figure 1A). Only 24% (12 of 50) of persons from disease-endemic regions had values greater than the two standard deviation cut-off value compared with the Indian NEC group.

To investigate whether cross-reactive antigens to the competing human-biting sand fly *P*. *papatasi* interfered with the *P*. *argentiptes* ELISA, we re-examined the sera with a *P*. *papatasi* saliva ELISA. This test confirmed the presence of antibodies to saliva of this sand fly; 36% (18 of 50) of sera of persons from leishmaniasis-endemic regions had IgG levels greater than those of the non-endemic control cut-off value (*P* = 0.0004) (Figure 1B). Moreover, a positive correlation between IgG levels against the saliva from both these human-biting sand flies was present in persons in India from VL-endemic areas (*P* < 0.0001, Spearman r = 0.71) (Figure 1D). These data suggest that persons from VL-endemic regions are being bitten by *P*. *papatasi* and *P*. *argentipes* to a similar extent.

To improve the specificity of the ELISA for detection of *P*. *argentipes*, we tested the utility of a serum pre-adsorption step against *P*. *papatasi* saliva. Re-examination of the human sera with the modified *P*. *argentipes* ELISA method (Figure 1C) resulted in a greater degree of separation between the non-endemic and disease-endemic groups from India (*P* < 0.0001), with 82% (51 of 62) of sera from persons from VL-endemic regions above the cut-off value.

**Figure 2.**
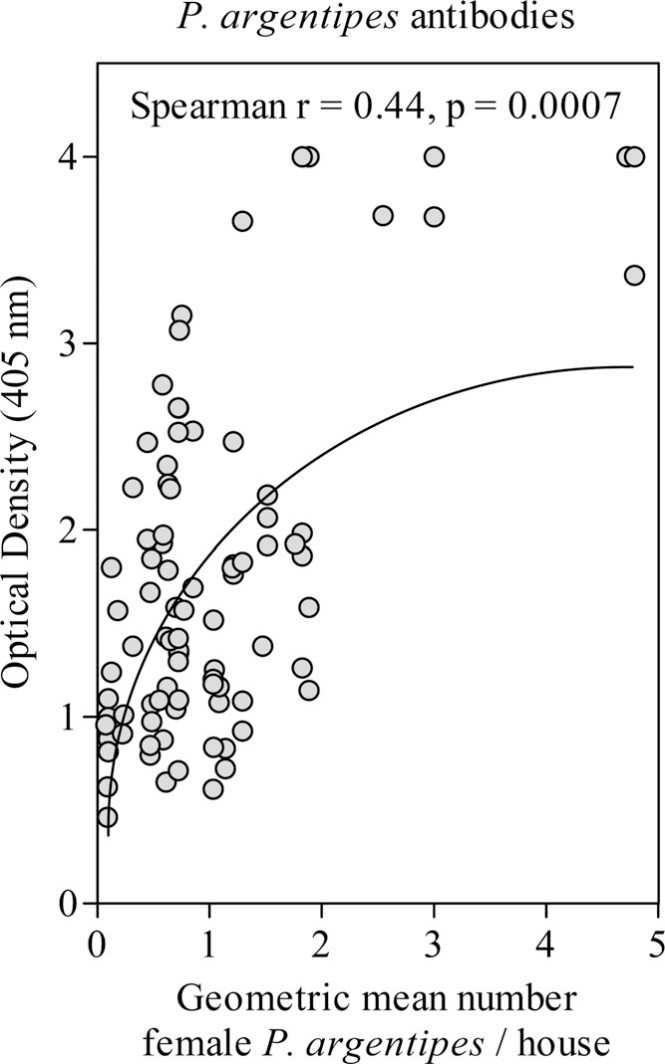
Correlation of human IgG levels to *Phlebotomus argentipes* saliva with indoor sand fly densities. Forty-three Indian and Nepalese households from five control clusters were surveyed for 15 months for densities of *P. argentipes*. Sand flies were sampled overnight by using CDC light traps. At the end of the survey, sera from all residents (n = 87) was collected, assayed for antibodies against *P. argentipes* saliva by using the *P. papatasi* pre-adsorbed enzyme-linked immunosorbent assay method and compared with average peak season *P. argentipes* indoor densities.

To test the ability of the *P*. *argentipes* saliva ELISA to measure sand fly exposure in the field, we conducted entomologic surveys of 28 households in India and 15 households in Nepal in VL-endemic foci. Using the *P*. *papatasi* pre-adsorbed saliva ELISA, we observed a significant logarithmic correlation between the antibody responses against *P*. *argentipes* saliva in sera from persons in India and Nepal sera and the 15-month geometric average number of indoor female *P*. *argentipes* trapped per household (Spearman r = 0.44, *P* = 0.0007) (Figure 2). A similar result was also found with antibodies against *P*. *papatasi* saliva (Spearman r = 0.44, *P* < 0.0001, data not shown). Analysis of individual clusters for geometric mean peak sand fly densities (recorded during October and November 2006) and saliva antibody responses confirmed that there was no bias between clusters (Supplementary Table 1, available at www.ajtmh.org).

**Figure 3.**
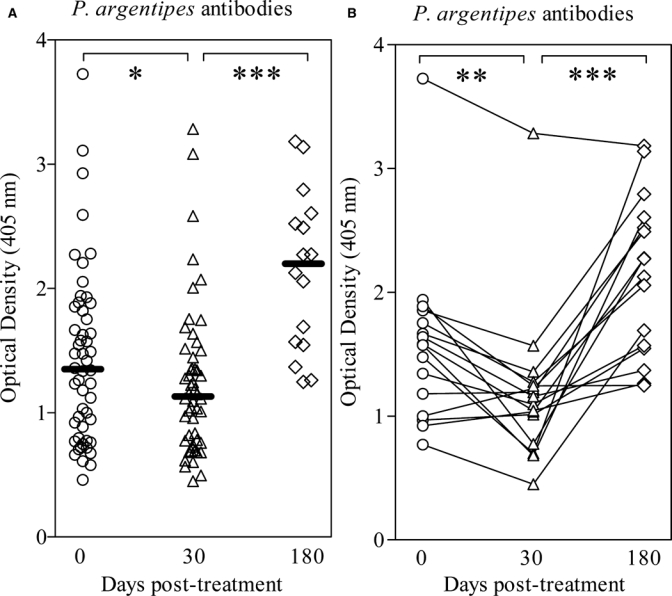
Decrease in human IgG levels to *Phlebotomus argentipes* saliva after vector intervention. **A**, Sera were analyzed for antibodies against the saliva of *P. argentipes* (using the *P. papatasi* pre-adsorbed enzyme-linked immunosorbent assay method) from patients with visceral leishmaniasis taken at the point of diagnosis (open circles), after their 30-day treatment in hospital (open triangles) and 6 months after their discharge (open diamonds) (n = 52). During their stay in the hospital, patients were protected from further exposure from sand fly bites with untreated bed nets. **B**, Paired antibody levels for a subsample of 16 patients. Bars represent the median value of the groups. Asterisks indicate statistical significance between the groups indicated (ns = not significant; *P < 0.05; **P < 0.005; ***P < 0.0005).

To be considered a useful tool for measurement of recent exposure to sand fly bites, the antibody response of humans to sand fly saliva needs to be transient. To observe the kinetics of antibody responses against *P*. *argentipes* saliva in humans during vector intervention, we monitored VL patients immediately before hospitalization (day 0), at the end of their course of drug treatment (day 30), and six months after their discharge (day 180) (Figure 3). During hospitalization, patients were screened from additional sand fly bites by the use of untreated bed nets. Despite the wide variation, using the pre-adsorbed saliva ELISA, we found that the median antibody titers to *P*. *argentipes* saliva decreased during their stay in a hospital (day 0 versus day 30: *P* = 0.010) (Figure 3A). However, antibody levels then increased to greater than pre-hospitalization levels by six months after discharge (day 30 versus day 180, *P*. *argentipes*; *P* < 0.0001). Pairwise analysis of 16 sera for which all three time points were collected (Figure 3B) confirmed a decrease in individual saliva antibodies when persons slept under untreated bed nets (day 0 versus day 30; *P* = 0.0007). A similar response was also seen for antibody levels against *P*. *papatasi* saliva, with a moderate decrease from day 0 to day 30 (*P* = 0.19), followed by a significant increase after release from a hospital (*P* = 0.001). From these data, we calculated that for people in India, half-life of IgG against *P*. *argentipes* saliva is approximately 166 days.

## Discussion

We report the human antibody response to saliva of *P*. *argentipes*, the vector of VL in the Indian sub-continent. In human populations from VL-endemic areas, we found that levels of IgG reflect differences in sand fly exposure.

Sand fly saliva is a complex mixture of pharmacologically active compounds whose combined function is to help the sand fly obtain blood during a bite. This saliva is known to contain a variety of anti-hemostatic, vasodilatory, and immunomodulatory compounds.^13^ Therefore, saliva will always be present during a bite from either an uninfected or infected sand fly. Sand fly saliva is also highly immunogenic,^9^–^12^,^14^ and antibodies against saliva of specific sand fly vectors have been valued as potential tools for measurement of community or personal exposure to sand fly bites by ELISA and risk of *Leishmania* infection. The complexity of sand fly saliva is such that different species, and perhaps sibling species, of sand flies contain specific antigens,^14^,^15^ which reinforce the utility of this approach. It is expected that any salivary antigen used for this purpose should be 1) specific for the bite of the vector species in question, 2) sensitive enough to use as a measure of relative exposure, and 3) exhibit low persistence in the host to accurately reflect changes in vector exposure with time.

Our pre-adsorbed *P*. *argentipes* saliva ELISA could distinguish persons from a sand fly–free country (United Kingdom) or persons from urban areas of India where VL is rarely reported from those sera collected from persons in the VL-endemic State of Bihar, India. Pre-adsorption also enhanced the ability of the ELISA to distinguish between the Indian non-endemic control and VL endemic groups. The exact reason for this finding is unknown, but one explanation may be the result of the presence of *P*. *papatasi* and absence (or much lower frequency) of *P. argentipes* sand flies in the NEC region of Western Uttar Pradesh. More in depth entomologic surveying of Indian NEC regions are required to address this possibility. In India, *P*. *argentipes* and *P*. *papatasi* are the most frequent human-biting sand flies. Exploratory experiments using mice bitten by either *P*. *papatasi* or *P*. *argentipes* colonized sand flies showed that cross-reactive antibodies exist and that inclusion of a *P*. *papatasi* saliva pre-adsorption step significantly reduced their contribution in an ELISA for detection of antibodies against *P*. *argentipes* saliva (Hostomska J, Volf P, Rogers MR, unpublished data). Using this assay with human sera, we significantly reduced levels of cross-reactive antibodies against saliva between these sand fly species and vastly improved the specificity of the ELISA towards the VL vector. In addition, a useful byproduct of the pre-adsorption method is the ability to measure the antibody response to the competing sand fly species (*P*. *papatasi*).

To test the sensitivity of the *P*. *argentipes* saliva ELISA to reflect differing amounts of exposure to this sand fly, we surveyed households in India and Nepal for the number of *P. argentipes* and *P*. *papatasi* present over a 15–16-month period. Houses were selected from control clusters of the KALANET program in which no insecticide-treated bed nets were used. This survey showed that the average household sand fly densities of *P*. *argentipes* and *P*. *papatasi* showed a strong correlation with the antibody response of the residents to these endophilic and endophagic sand flies. It appears that the sensitivity of the assay is reduced when the *P*. *papatasi* saliva pre-adsorption step was included, as shown by the Indian NEC group. However, the overall benefit of this modification is the increased its specificity towards the VL vector. The specificity and sensitivity of the assay may be further improved if whole *P*. *argentipes* saliva could be replaced by recombinant antigen(s) that have been screened for their ability to reflect specific exposure to this fly bite. Because this approach will not require labor-intensive sand fly rearing and salivary gland dissection, this modification will make the assay more amenable for large-scale application.

Finally, we addressed the ability of antibodies against *P*. *argentipes* saliva to identify recent periods of non-exposure to sand fly bites. Such a tool would only have value for monitoring vector control measures that reduce sand fly contact rates for humans if antibody titers are transient and increase relatively quickly after re-exposure. This potential use has been observed for mosquito and tick salivary antigens in humans^16^,^17^ and triatomine salivary antigens in mice.^18^ To our knowledge, no studies have tested the kinetics of antibodies in humans against sand fly saliva from the field, but laboratory experiments with naive dogs exposed to the bites of the sand fly *Lutzomyia longipalpis* have found significant decreases in IgG against sand fly saliva and IgG2 titers within weeks after exposure.^19^ In the present study, we show that VL patients hospitalized and protected from sand fly bite by untreated bed nets was corroborated by an average 15% reduction of antibody titers against *P*. *argentipes* after 30 days. Upon discharge from hospitals, antibody levels against saliva in these patients increased significantly beyond their original pre-treatment levels after six months re-exposure to bites. This finding indicates a classic antibody memory response to saliva of both species of sand flies.

Thus, the *P*. *argentipes* saliva ELISA is likely to be useful in understanding the epidemiology of VL. For example, *L. donovani* has been identified in the blood of domesticated animals sampled from VL-endemic regions of Nepal (Dujardin JC, unpublished data), and the saliva ELISA could help investigate this possible zoonotic cycle of transmission. A study in children in Brazil in whom antibodies against *Lutzomyia longipalpis* sand fly saliva developed showed that they were more likely to resist zoonotic VL caused by *L*. *infantum chagasi*.^9^,^10^ Whether a similar effect occurs between *P*. *argentipes* saliva and *L*. *donovani* infection in the Indian subcontinent remains to be tested but the assay we describe should be instrumental in these studies.

The novel saliva ELISA we describe is a significant step towards determining the communal (and eventually personal) level of *P*. *argentipes* exposure in human populations at risk for VL. After validation on a larger scale, this method will improve evaluation of vector control programs and enable investigation of new aspects of the epidemiology of this neglected tropical disease.

## Supplementary Material

Supplementary Table 1: Comparison of Indian and Nepalese household sand fly densities and anti-saliva antibody levels. During the KALANET study sand flies were sampled monthly by CDC light trap for 15 months in households from control clusters (i.e. no bed nets and no insecticide house spraying) from VL endemic areas. Peak season sand fly densities taken from October and November 2006 are shown. Sera from the household residents were taken at the end of the survey and analyzed for *P. argentipes* and *P. papatasi* anti-saliva responses by ELISA. For comparison, the average non-endemic control anti- *P. argentipes* salivary antibody O.D. during this time was 0.7 ± 0.2.
