# Supplementary material for: Measurement of Recent Exposure to Phlebotomus argentipes, the Vector of Indian Visceral Leishmaniasis, by Using Human Antibody Responses to Sand Fly Saliva
Source: Am J Trop Med Hyg. 2010 May;82(5):801–7. doi: 10.4269/ajtmh.2010.09-0336 (PMC2861389; doi:10.4269/ajtmh.2010.09-0336)
Supplement: Supplementary Table 1: Comparison of Indian and Nepalese household sand fly densities and anti-saliva antibody levels. During the KALANET study sand flies were sampled monthly by CDC light trap for 15 months in households from control clusters (i.e. no bed nets and no insecticide house spraying) fro [file SD2.pdf]

Am. J. Trop. Med. Hyg., 82(5), 2010, p. 1
doi:10.4269/ajtmh.2010.09-0336
Copyright © 2010 by The American Society of Tropical Medicine and Hygiene

Supplementary Table 1

Comparison of Indian and Nepalese household sand fly densities and anti-saliva antibody levels. During the KALANET study sand flies were sampled monthly by CDC light trap for 15 months in households from control clusters (i.e. no bed nets and no insecticide house spraying) from VL endemic areas. Peak season sand fly densities taken from October and November 2006 are shown. Sera from the household residents were taken at the end of the survey and analyzed for *P. argentipes* and *P. papatasi* anti-saliva responses by ELISA. For comparison, the average non-endemic control anti-*P. argentipes* salivary antibody O.D. during this time was 0.7 ± 0.2.

				Household sand fly density (Geometric mean ± SD)		Household anti-saliva antibody titre (Av. O.D. ± SD)	
Cluster	Country	Houses	People/ house	*P. argentipes*	*P. papatasi*	*P. argentipes*	*P. papatasi*
C05	India	8	13	0.87 ± 0.46	0	1.87 ± 0.39	1.42 ± 0.50
C06	India	10	19	0.84 ± 0.47	0.008 ± 0.03	1.58 ± 0.83	1.63 ± 0.56
C09	India	10	26	1.08 ± 0.69	0	1.09 ± 0.81	1.32 ± 0.55
C55	Nepal	9	14	0.58 ± 1.03	0.02 ± 0.04	1.32 ± 1.03	1.67 ± 0.61
C56	Nepal	6	15	2.06 ± 2.48	1.27 ± 1.55	1.16 ± 0.71	2.97 ± 0.89
